# Supplementary material for: Structural Entities Associated with Different Lipid Phases of Plant Thylakoid Membranes—Selective Susceptibilities to Different Lipases and Proteases
Source: Cells. 2022 Aug 28;11(17):2681. doi: 10.3390/cells11172681 (PMC9454902; doi:10.3390/cells11172681)
Supplement: Supplementary file 1 [file cells-11-02681-s001.zip › cells-1860496-supplementary.pdf]

Structural entities associated with different lipid phases of plant thylakoid membranes – selective susceptibilities to different lipases and proteases

Ondřej Dlouhý, Václav Karlický, Uroš Javornik, Irena Kurasová, Ottó Zsiros, Primož Šket, Divya Sai Kanna, Kinga Böde, Kristýna Večeřová, Otmar Urban, Edward S. Gasanoff, Janez Plavec, Vladimír Špunda, Bettina Ughy, Győző Garab

Supplementary material:

Figure S1. ^{31}P-NMR spectrum of untreated spinach thylakoid membranes and the fitted spectrum obtained after the deconvolution of the spectral components.

Figure S2. ^{31}P-NMR spectra of spinach thylakoid membranes treated with 24 U mL^{-1} PLA1.

Figure S3. Variations of the total psi-type CD amplitude in the Q_y region of 2 U mL^{-1} PLA1-treated thylakoid membranes

Figure S4. Effects of 5 U mL^{-1} WGL treatments on different biophysical and biochemical properties of isolated spinach thylakoid membranes.

Figure S5. Deconvoluted ^{31}P-NMR spectral components of isolated Trypsin-treated spinach thylakoid membranes before and after the irradiation of the sample with satutrating pulses.

Figure S6. Effects of 10 mg mL^{-1} Trypsin treatments on different biophysical and biochemical parameters of isolated spinach thylakoid membranes.

Figure S7. Effects of 16 U mL^{-1} Proteinase K treatments on different biophysical and biochemical parameters of isolated spinach thylakoid membranes.

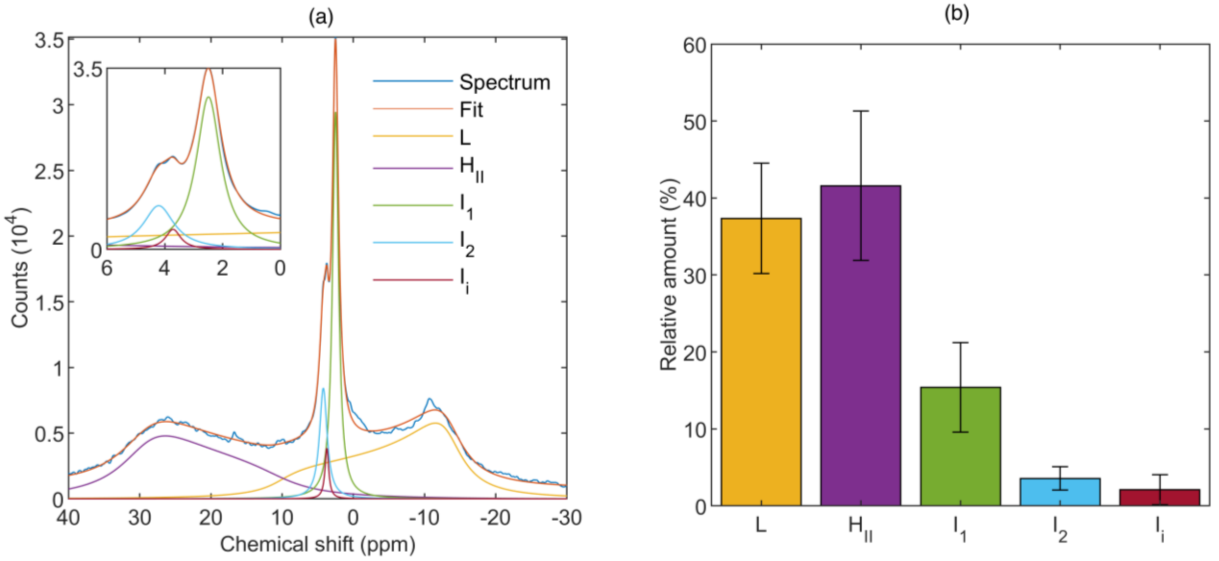

Figure S1. (a) ^{31}P-NMR spectrum of untreated spinach thylakoid membranes (dark blue trace) and the fitted spectrum (orange curve) obtained after the deconvolution of the spectral components. The measured spectrum represents the average obtained from 14 independent experiments performed at 5 °C, each with 15 min acquisition time on freshly isolated untreated samples which were used as controls for different lipase and protease treatments; the average Chl content of these samples was 8.3 ± 2.9 mg mL^{-1}. Individual contributions of the lamellar (L, yellow), inverted hexagonal (H_{II}, purple), and isotropic (I_1, I_2 and I_i, green, light blue and red, respectively) lipid phases were determined using the software DMfit. Inset shows the isotropic region. (b) Integrated areas of the deconvoluted component spectra, associated with the different lipid phases, relative to the overall integrated area; mean values ± SD, n = 14.

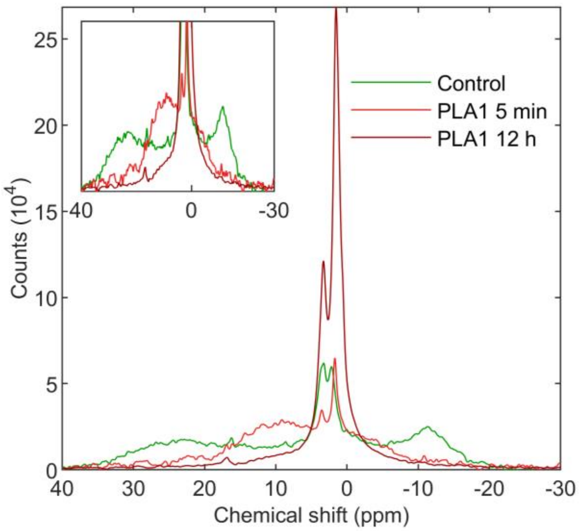

Figure S2. ^{31}P-NMR spectra of untreated spinach thylakoid membranes (green; acquisition time, 1 h) and treated with 24 U mL^{-1} PLA1 for 5 min (light red; acquisition time, 5 min) and 12 h (dark red; acquisition time, 1 h); temperature, 5 °C. The spectra are normalized to total Chl contents 10 mg mL^{-1} and total acquisition times of 15 min. Inset highlights the L and H_{II} regions.

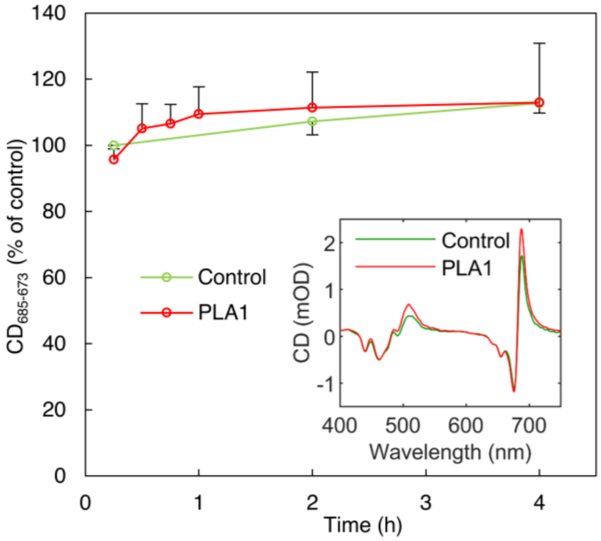

Figure S3. Variations of the total psi-type CD amplitude in the Q_y region, (+)685 – (-)673, of isolated spinach thylakoid membranes, as a function of time, in the control (green) and in samples treated with 2 U mL^{-1} PLA1 (red); temperature, 5 °C. Inset shows typical CD spectra of control (green) and samples treated with 2 U mL^{-1} PLA1 for 2 h (red).

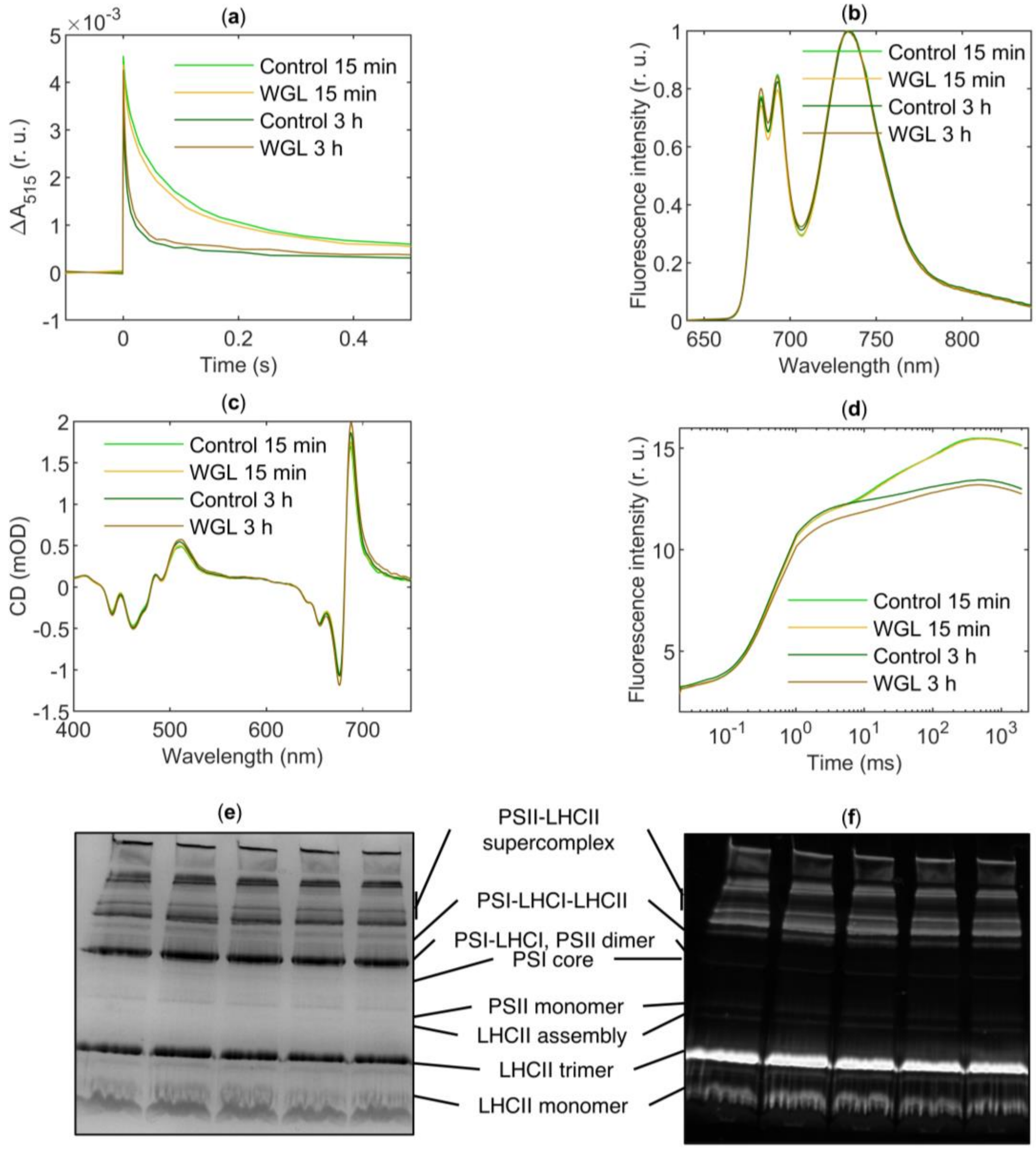

Figure S4. Effects of 5 U mL^{-1} WGL treatments of different lengths (as indicated) on different biophysical and biochemical properties of isolated spinach thylakoid membranes: (a) ΔA_{515} kinetics, (b) 77 K Chl-*a* fluorescence emission spectra, (c) CD spectra, (d) OJIP transients and (e and f) CN-PAGE transilluminated with visible light and Chl-*a* fluorescence, respectively; lanes left to right: control and samples solubilized after 0, 30, 60 and 120 min incubation in the presence of WGL; temperature, 5 °C. Typical traces and spectra are shown.

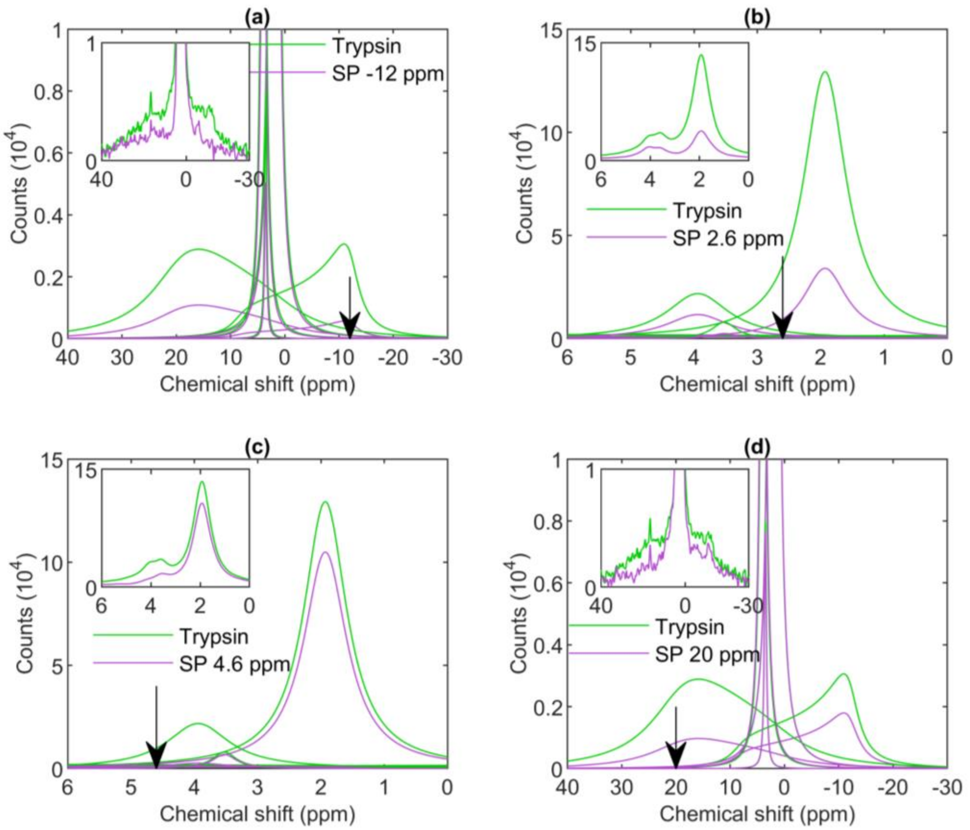

Figure S5. Deconvoluted ^{31}P-NMR spectral components of isolated Trypsin-treated spinach thylakoid membranes before and after the irradiation of the sample with satutrating pulses. The pulses were applied at different frequencies, as indicated by the arrows, at or close to the peak positions of different phases: (a) L, -12 ppm; (b) the breakdown product, 20 ppm; (c) I_1, 2.8 ppm; and (d) I_2, 4.3 ppm. Each panel shows the deconvoluted component spectra and, in inset, the measured spectra between 40 and -30 ppm (a,d) and 6 and 0 ppm (b,c) of Trypsin-tretated before (green) and after (purple) the saturation pulse. Typical spectra are shown; temperature, 5 °C. The samples were treated for 4 h at 5 °C with 10 mg mL^{-1} Trypsin.

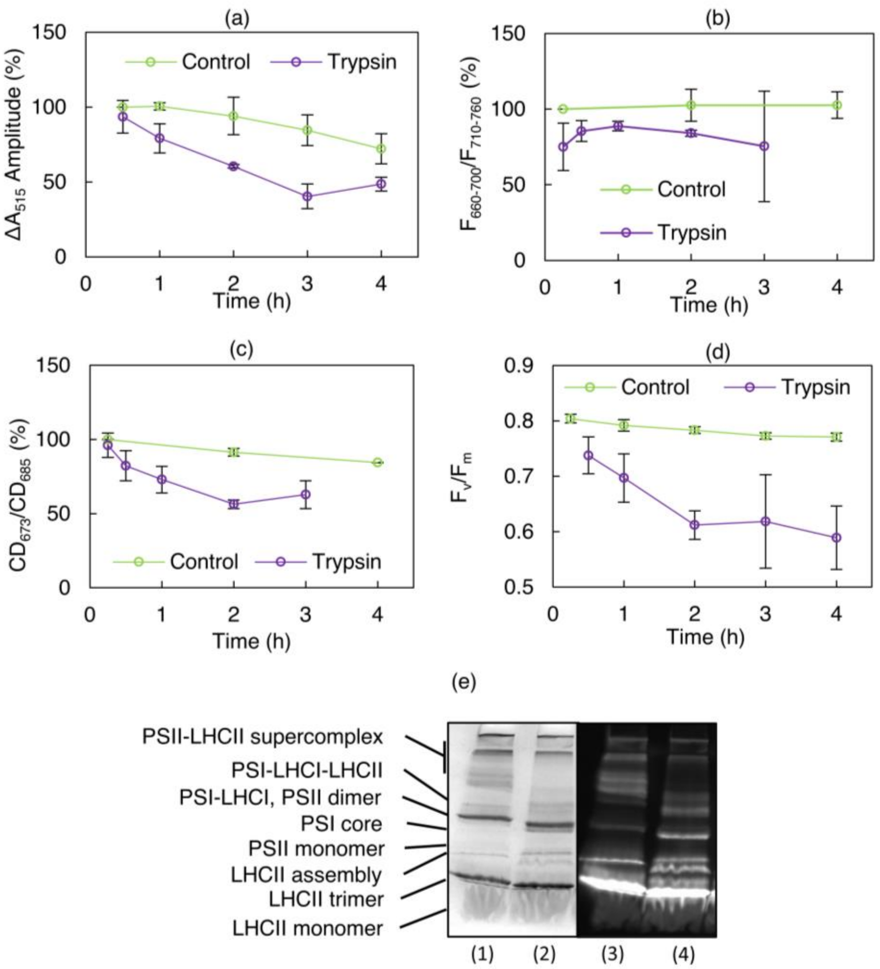

Figure S6. Effects of 10 mg mL^{-1} Trypsin treatments of different lengths (as indicated) on different biophysical and biochemical parameters of isolated spinach thylakoid membranes: (a) initial amplitudes of ΔA_{515}; (b) ratio of the integrated fluorescence intensities $F_{660-700}/F_{710-760}$; (c) ratio of the amplitudes of the (-)673/(+)685 CD bands; and (d) F_v/F_m Chl-*a* fluorescence parameter. Mean values and standard deviations from three independent experiments; in (a-c), values relative to the untreated controls. (e) CN-PAGE transilluminated with visible light (left) and Chl-*a* fluorescence (right), lanes (1) and (3): control; (2) and (4): treated for 1 h; temperature, 5 °C.

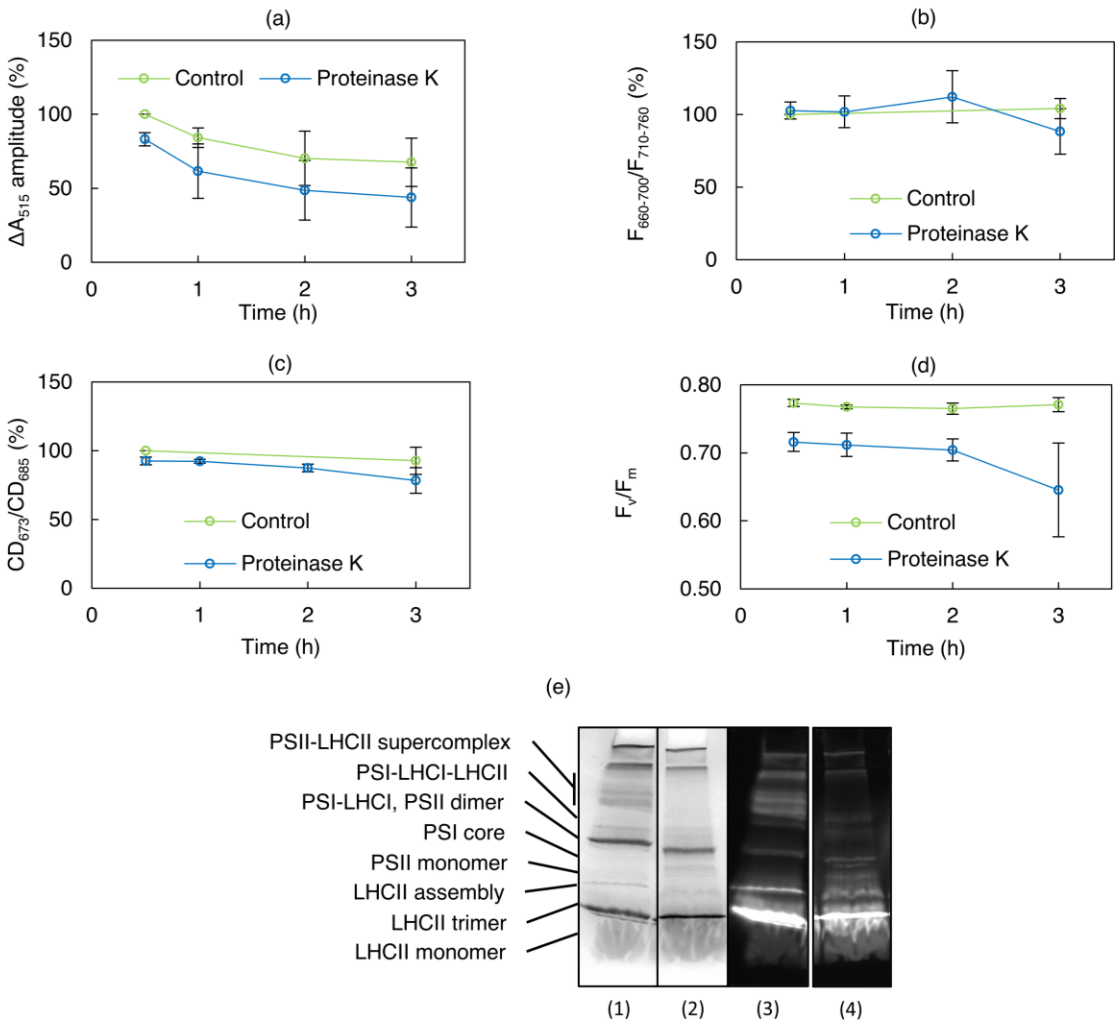

Figure S7. Effects of 16 U mL^{-1} Proteinase K treatments of different lengths (as indicated) on different biophysical and biochemical parameters of isolated spinach thylakoid membranes: (a) initial amplitudes of ΔA_{515}; (b) ratio of integrated fluorescence intensities $F_{660\text{-}700}/F_{710\text{-}760}$; (c) ratio of the amplitudes of the (-)673/(+)685 CD bands; (d) F_v/F_m Chl a fluorescence parameter. Mean values and standard deviations from three independent experiments. (e) CN-PAGE transilluminated with visible light (lanes (1) and (2)) and Chl-*a* fluorescence (lanes (3) and (4)); lanes (1) and (3): control; (2) and (4): loaded on gel 1 h after the 30 min Proteinase K treatment at 22 °C. Temperature of all measurements, 5 °C.
